# Optimization of fermentation conditions for *Metarhizium robertsii* and its effect on rice growth

**DOI:** 10.1128/spectrum.01009-26

**Published:** 2026-09-24

**Authors:** Xiaoyuan She, Deling Wang, Meng Zhu, Jiaolong Fu, Dongfei Han, Jing Bai

**Affiliations:** 1 School of Chemistry and Life Sciences, Suzhou University of Science and Technology 66339, Suzhou, China; 2 School of Environmental Science and Engineering, Suzhou University of Science and Technology 66339, Suzhou, China; Institute of Eco-environmental and Soil Sciences, Guangzhou, Guangdong, China

**Keywords:** *Metarhizium robertsii*, *Oryza sativa*, fermentation optimization, growth-promoting mechanism, endophytic fungal community

## Abstract

*Metarhizium robertsii* ARSEF 23 is a significant insect-pathogenic fungus and plant-associated endophyte, with dual roles as an insect-control agent and plant growth-enhancing agent that have fueled its extensive use in agricultural practices. However, the growth-promoting impacts of *M. robertsii* (referred to as MAA) on rice, and their underlying mechanisms are still not fully clarified. This research verified the growth-enhancing effects of both MAA itself and its fermented supernatant on rice plants. By means of single-factor tests and response surface methodology, the fermentation conditions of MAA were then optimized using rice root length as the primary index, while the possible mechanisms driving this growth promotion were initially explored. The results showed that both MAA and its fermented supernatant exhibited notable growth-enhancing impacts on rice. Sucrose and peptone were found to be the best carbon and nitrogen substrates, respectively. The most remarkable growth promotion was observed when the fermented supernatant—obtained under conditions of pH 6.0, 0.2% β-Ala, and 100 μmol/L aluminum sulfate—was applied, leading to a 21.7% rise in rice root length relative to the control group, which further has an impact on the metabolites of MAA. In addition, the relative abundance of MAA increased by 400-fold in rice roots, strongly suggesting root association, and significantly restructured the root endophytic fungal community by suppressing pathogenic genera (e.g., *Fusarium* sp.) and enriching plant growth-promoting beneficial genera (e.g., *Trichoderma* sp.). Meanwhile, marked increases in chlorophyll content (a 28.17% rise compared to the control) and more abundant root hairs were also observed in rice treated with the MAA fermented supernatant. Collectively, these hydroponic-based findings provide a theoretical basis for the development and utilization of environmentally friendly and high-efficiency MAA-derived bio-inoculants in rice production, though field validation is still needed.

**IMPORTANCE:**

*Metarhizium robertsii* is a promising entomopathogenic and endophytic fungus with biocontrol and plant growth-promoting functions. Its growth-promoting effects and mechanisms on rice remain poorly understood, and fermentation conditions tailored for rice have not been optimized. This study optimized fermentation parameters, revealed the growth-promoting mechanism, and provided a technical basis for developing green, efficient microbial inoculants to support sustainable rice production.

## INTRODUCTION

*Metarhizium robertsii* ARSEF 23, a notable group of biocontrol fungi with a broad insect host range, is widely used in agriculture (1). Incomplete statistics indicate that over 200 insect species worldwide can be infected and killed by this particular fungus. With the deepening of research, it has been found that in addition to infecting and destroying target pests, *M. robertsii* can also associate with plant roots. Acting as an endophyte, it further aids in boosting plant growth (2, 3). Therefore, as a microbial resource with a wide range of application prospects, the role of *M. robertsii* in promoting the growth and development of plants has attracted increasing attention. *M. robertsii* forms symbiotic relationships with agriculturally and economically vital plants, including tomato, Arabidopsis, soybean, maize, and wheat (4–7). When *M. robertsii* associates with plants, it can provide benefits such as growth promotion (8–10), facilitating nutrient transfer (11), and protection against pests (9, 10) and diseases (12, 13). For example, tomato plants hosting *M. robertsii* showed marked increases in root length and overall height compared to non-inoculated control groups (14). When Arabidopsis was inoculated with three *Metarhizium anisopliae* strains (Ma-20, Ma-25, and Ma-28), its fresh weight and total chlorophyll content rose significantly after 7 days. The primary root length was promoted, and β-caryophyllene and o-cymene were identified as key metabolites during this interaction. However, research on the plant growth-promoting effect of *M. robertsii* remains immature, with the scope of researched plants limited. Especially, there are only a few reports on the interaction between *M. robertsii* and rice, and these existing studies solely focus on rice resistance to heavy metals and salt stress (15–17). The endophytic and growth-promoting effects of *M. robertsii* on rice remain unclear.

The production of secondary metabolites plays a key role in the plant growth-promoting effect. For example, pipecolic acid (18) can form a non-selective rhizosphere relationship between *M. robertsii* and *Arabidopsis thaliana*. After *M. robertsii* inoculation, Arabidopsis shows elevated pipecolic acid levels, which strengthen its resistance to plant pathogens and aphids (18). When four *M. robertsii* strains were co-cultured with soybean and corn, destruxins (DTXs)—including 25 DTX analogues—were detected. Notably, DTX yields varied significantly depending on the associated plant species (19). Kumar showed that *Metarhizium* can produce a range of organic acids (e.g., keto-gluconic acid, oxalic acid) that promote zinc solubilization, as well as secondary metabolites such as indole-3-acetic acid (IAA) and iron carriers that promote the growth of rice and cardamom plants (20). Notably, the output of these bioactive secondary metabolites depends not only on the fungus’s inherent genetic traits but also on factors like culture media, nutrient composition, and fermentation conditions (21). Optimizing the physical and chemical factors influencing fermentation—such as temperature, pH, aeration, and medium components—is essential for maximizing the yield of specific biological products (22). By refining medium composition and fermentation parameters, the production of growth-promoting secondary metabolites can be significantly boosted (23). He et al. showed that optimal fermentation conditions of *M. robertsii* increased the inhibition rate of *Fusarium solani*, the main pathogenic fungus causing the root rot of wolfberry (*Lycium barbarum*) (24). Using response surface methodology (RSM), a maximum indole auxin production of 208.3 ± 0.4 mg IAA eq/L by *Pantoea agglomerans* was achieved, which represented a 40% increase compared with the growth conditions used in previous studies (25). RSM is widely used for fermentation optimization. Additionally, there are few studies focusing on optimizing secondary metabolite production in *M. robertsii* specifically for plant growth promotion, and no reports on the optimization of *M. robertsii* fermentation conditions targeting rice growth promotion to date.

Given these gaps, this study aimed to (i) analyze the effects of carbon/nitrogen sources and fermentation conditions on *M. robertsii*’s growth-promoting activity; (ii) optimize these conditions via RSM; and (iii) explore the underlying growth-promoting mechanisms in rice.

## MATERIALS AND METHODS

### Fungal strain, culture medium, and plant

#### *Metarhizium* and rice experiment materials

Prof. Yuquan Xu of the Biotechnology Research Institute, Chinese Academy of Agricultural Sciences, generously supplied the fungus *Metarhizium robertsii* ARSEF 23 (hereafter referred to as MAA). To harvest fresh conidia, this fungal strain was grown on potato dextrose agar plates (BD Difco) and incubated at 28°C for a 7 day period. For the purpose of preparing MAA fermentation broth to assess its rice growth-promoting effects, potato dextrose broth (PDB) medium (BD Difco) was used; additionally, the fermentation conditions of MAA were optimized with a basic fermentation medium. Said basic medium consisted of the following components: 6 g peptone, 0.5 g K₂HPO₄, 10 g glucose, 0.5 g KH₂PO₄, 0.01 g MgSO₄, and distilled water to make a total volume of 1 L. Various carbon and nitrogen sources were employed to substitute for glucose and peptone in this basic medium.

The experimental material comprised *Oryza sativa L*. Shuhui 527 (SH527) seeds, a prominent indica hybrid rice restorer line in China.

#### Reagents for molecular analysis

Phenol, chloroform, isoamyl alcohol, sodium acetate (NaAc), isopropanol, ethanol, proteinase K, and TE buffer were purchased from Sangon Biotech (Shanghai) Co., Ltd., China. Primers *ITS1* and *ITS4* were synthesized by Sangon Biotech (Shanghai) Co., Ltd. The 2×Easy Taq PCR Supermix was obtained from Vazyme Biotech Co., Ltd., Nanjing, China. All reagents were of molecular biology grade.

### Determination of the growth promotion effect of MAA strain on rice

Transfer 1 mL of spore suspension of MAA strain (1 × 10^7^ spores/mL) into 100 mL of PDB medium in 250 mL flasks and incubate the culture at 28°C, 220 rpm for 7 days. The post-fermentation broth was centrifuged at 4°C and 8,000 rpm for 5 min to obtain the supernatant, which was then sterilized through a 0.22 µm filter and stored at 4°C for further use.

Plump, uniform SH527 rice seeds were surface-sterilized with 75% alcohol and 10% NaClO (three sterile water rinses), soaked at 28°C for 8 h, and germinated on sterile filter paper in the dark for 3 days. Seedlings with consistent germination (coleoptile length: 1.0 ± 0.2 cm) were selected. An opaque cultivation system was established using black polyethylene hydroponic boxes (specifications: 126 × 87 × 113 mm, 1 L), and 96-well plates were used for seedling planting. Hoagland’s nutrient solution served as the hydroponic medium for rice growth. In the first 3 days after planting, 1/2-strength nutrient solution was applied; from the 4th day onward, it was replaced with full-strength solution, with the liquid level maintained to cover 2/3 of the root system. Sterile water was added every 48 h to make up for evaporative loss, and the nutrient solution was completely renewed every 72 h, with the pH adjusted to 5.8 ± 0.2. The treatment group received 3% sterilized MAA fermentation supernatant, while the control group was supplemented with an equal volume of sterile PDB medium. Each group had three biological replicates, and all were cultured under conditions of 16 h light/8 h dark photoperiod and 28°C. Seven days after treatment, the shoot height of rice plants was measured. For root length determination, the root systems were gently rinsed and straightened first, then the length of the longest root was measured with a ruler.

### Root association and endophytic community analysis

To investigate the effects of MAA on the endophytic fungal community in roots, a separate pot experiment was conducted. Germinated SH527 rice seedlings were transplanted into pots filled with a substrate composed of non-sterilized nutrient soil, perlite, and vermiculite (purchased from Shanghai A Stone Home Company) (2:1:1, v/v/v; 2 kg per pot). The nutrient soil was not sterilized to retain its native microbial community. The treatment group (M+) was inoculated with MAA spore suspension to a final concentration of approximately 2.5×10⁵ spores/g substrate, while the control group (M-) received an equal volume of sterile water. Each treatment had three replicates. After 30 days of greenhouse cultivation, the roots were harvested and surface-sterilized following these steps: rinsed with tap water → washed with sterile water → treated with 4% sodium hypochlorite for 5 min → washed with sterile water → immersed in 70% ethanol for 1 min → washed with sterile water → cut into pieces → washed with sterile water. Total root DNA was extracted using the phenol-chloroform method. The fungal ITS region was amplified with primers ITS1 and ITS4, and the PCR products were sequenced using an Illumina MiSeq system. The raw ITS and 16S rRNA sequencing reads have been deposited in the NCBI Sequence Read Archive (SRA) under BioProject accession number PRJNA1506420. Raw sequence data were processed via the QIIME2 workflow, encompassing quality control, denoising, and clustering into amplicon sequence variants (ASVs). Taxonomic classification was conducted with the UNITE database as reference. The relative abundance of each ASV was computed as its proportion relative to the total sequence count per sample. For bacterial community analysis, the V3–V4 hypervariable regions of the 16S rRNA gene were amplified using primers 338F (5′-ACTCCTACGGGAGGCAGCAG-3′) and 806R (5′-GGACTACHVGGGTWTCTAAT-3′). Amplicon sequencing was performed on an Illumina MiSeq platform (2 × 300 bp). Raw reads were processed through the QIIME2 pipeline, including quality filtering, denoising, and clustering into amplicon sequence variants (ASVs). Taxonomic assignment was performed using the SILVA database (v138). Bacterial community composition and relative abundances were analyzed using the same statistical methods as described for fungal communities. Each treatment had three biological replicates. Furthermore, due to limited biological replication for the pot experiments, multivariate analyses such as PCoA and PERMANOVA could not be reliably performed. This should be considered when interpreting the community-level comparisons.

### Effects of different culture media on MAA growth and rice growth promotion

Under the basic culture conditions consisting of glucose as the carbon source, peptone as the nitrogen source, pH 6.0, a cultivation period of 7 days, 0.2% β-alanine (β-Ala), and 50 μmol/L aluminum sulfate [Al₂(SO₄)₃], single-factor variable experiments were carried out with only one parameter adjusted at a time. The tested parameters were as follows: carbon source types (including glucose, sucrose, galactose, corn starch, ammonium sulfate, and soluble starch) to replace glucose in the basic medium; nitrogen source varieties (including yeast extract, L-glutamic acid, beef extract, KNO₃, and peptone) to substitute peptone in the basic medium; pH levels set at 5.0, 6.0, 7.0, 8.0, and 9.0; cultivation time levels of 7 days, 9 days, 11 days, 13 days, and 15 days; β-Ala concentration gradients of 0.1%, 0.2%, 0.3%, 0.4%, and 0.5%; and Al₂(SO₄)₃ concentration levels of 10 μmol/L, 20 μmol/L, 50 μmol/L, 100 μmol/L, and 150 μmol/L. The detailed composition of different culture media is presented in Table 1. All treatments were performed in triplicate and incubated in a rotary shaker at 220 rpm for 7 days.

**TABLE 1:** Effects of six factors and five horizontal medium factors on the growth of MAA and rice

Factor levels	Carbon source	Nitrogen source	Fermentation time (d)	β-Ala concentration (%)	Al_2_(SO_4_)_3_ supplementation (μmol/L)	pH
1	Glucose	Peptone	7	0.1	10	5.0
2	Soluble starch	Yeast	9	0.2	20	6.0
3	Corn flour	Glutamic acid	11	0.3	50	7.0
4	Galactose	(NH_4_)_2_SO_4_	13	0.4	100	8.0
5	Sucrose	KNO_3_	15	0.5	150	9.0

Fungal growth assessment: To investigate the effects of different culture media on fungal growth, solid media were prepared (compositions shown in Table 1). Each solid medium was poured into sterile Petri dishes and allowed to solidify. After solidification, 2 µL of conidial suspension (1 × 10⁷ spores/mL) was inoculated at the center of each Petri dish, followed by incubation at 28°C in the dark. Each treatment was set up with three replicates. Fungal growth was evaluated by measuring the colony diameter after 7 days of cultivation. Additionally, the morphological characteristics of the colonies (e.g., color, texture, and sporulation) were recorded.

Rice growth promotion assay: The growth-promoting effects of MAA cultured in different media (Table 1) on rice were evaluated using a hydroponic system with Hoagland nutrient solution. Each treatment included 96 rice seedlings. After 7 days of cultivation, the shoot height of rice plants was measured. Meanwhile, the root systems were gently rinsed and straightened, and the length of the longest root was measured with a straightedge at 7 days post-treatment as the root length index.

### Single-factor test of the effects of different fermentation conditions on the growth-promoting activity of MAA

Single-factor experiments were employed for optimization: under the basic experimental conditions outlined earlier, individual assessments were conducted on critical factors such as carbon source, nitrogen source, pH, incubation period, β-Ala concentration, and Al₂(SO₄)₃ supplementation. To comprehensively assess each variable, a set of 36 unique culture medium recipes was developed, and their complete component details are presented in Table S4. Growth-promoting efficacy was determined by comparing the shoot height of 7-day-old rice seedlings, as well as the shoot height and root length of 15-day-old seedlings.

### Response surface optimization test

**Fig 1:**
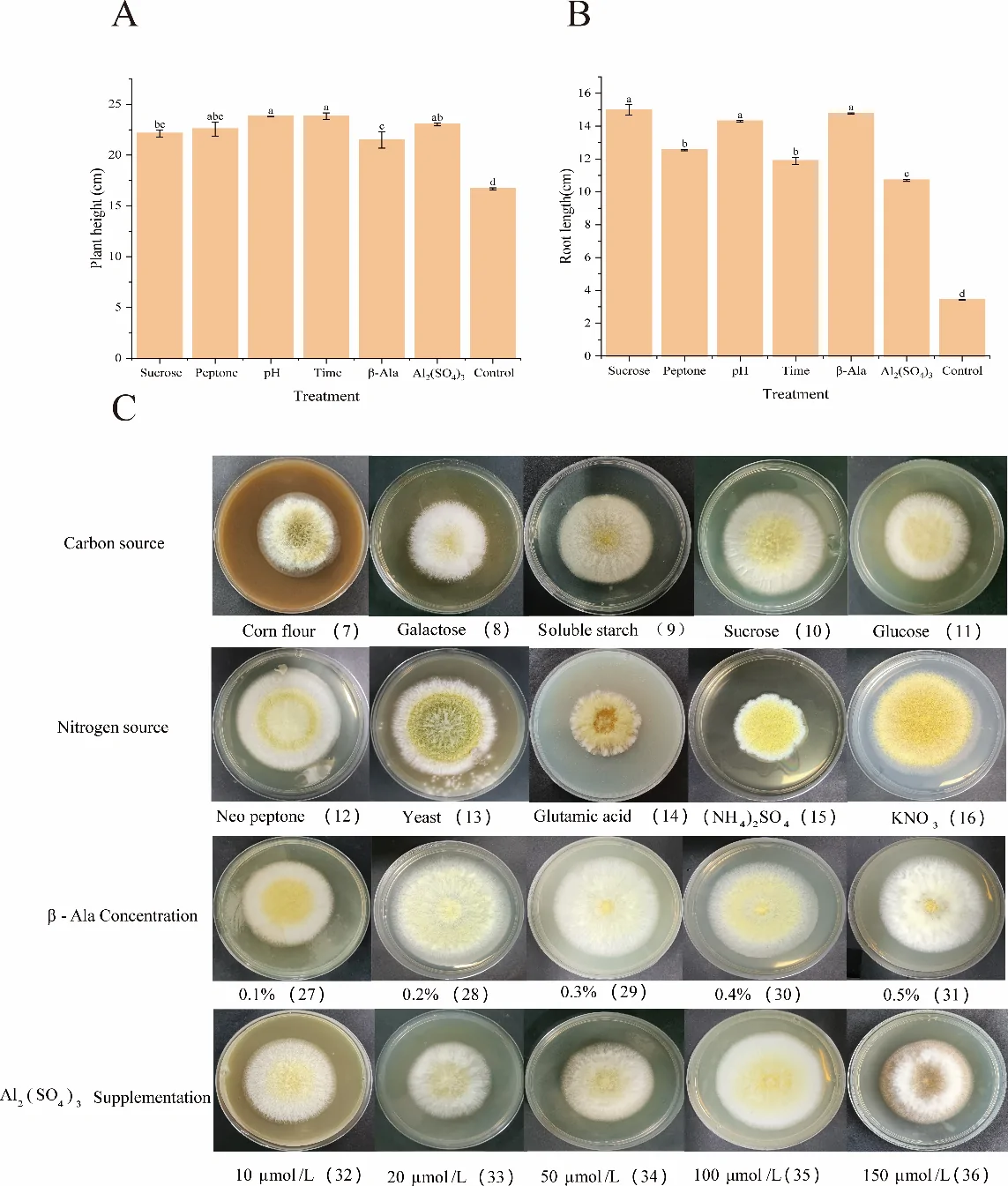
Effects of different culture conditions on rice seedling growth and the growth of MAA. (**A**) and (**B**), respectively, represent the effects of MAA fermentation culture in different liquid media on the plant height and root length of rice. Control indicates the culture of MAA in the basal medium, and treatment indicates the optimal value under the single-factor experimental design. The horizontal coordinates sucrose and peptone respectively represent the replacement of carbon and nitrogen sources in the basic culture medium, pH indicates the alteration of the pH value of the culture medium, time represents the change in the fermentation time of MAA, and β-Ala and Al_2_(SO_4_)_3_ respectively represent the addition of different concentrations of β-Ala and Al_2_(SO_4_)_3_; (**C**) effect of different culture methods on solid medium. The influence of different solid media on the growth of MAA. The numbers in parentheses indicate the numbering of culture media in Table S4.

Root length was selected as the primary response variable for RSM optimization because it is a sensitive and early indicator of plant growth promotion in hydroponic systems. Single-factor experiments (Fig. 1) showed that root length responded more consistently to fermentation condition changes than shoot height or biomass. Moreover, root length is non-destructively measurable and correlates with nutrient uptake capacity. Based on the single-factor test results, key parameters including (i) sucrose concentration, (ii) pH, (iii) β-Ala concentration, and (iv) Al₂(SO₄)₃ supplementation were further optimized. The RSM was employed following established protocols (24, 26), with the experimental design detailed in Tables S1 and S2. The optimization results were evaluated based on the 7 day root length of rice.

### Evaluation of rice growth-promoting traits

Additional growth-promoting characteristics were assessed, including chlorophyll content, adventitious root number, and root hair phenotype.

#### Chlorophyll content

The relative chlorophyll content was determined using a SPAD-502 Plus chlorophyll meter, with measurements taken at a standardized position on the leaves.

#### Adventitious root number

After 15 days of hydroponic cultivation, the adventitious roots were counted for each plant (*n* = 96). The adventitious root number was calculated as the average number of adventitious roots distributed along the crown root.

#### Root hair phenotype

To quantify root hair development, 7-day-old roots were imaged in the root maturation zone using an FV1000 confocal microscope (Olympus).

The growth rate of each parameter was calculated as growth rate (%) = [(treatment group value − control group value) / control group value] × 100.

### Statistical analysis

All experiments were performed with at least three biological replicates. Data are presented as means ± standard error (SE), and Origin 2022b was used for graphing (Origin Lab Corporation, Northampton, MA, USA). Statistical significance among treatments was determined by one-way ANOVA followed by Fisher’s Least Significant Difference *post hoc* test (*P* < 0.05) using SPSS 22.0 (IBM SPSS Statistics, Chicago, IL, USA). Significant differences between treatment groups are indicated by different superscript letters.

## RESULTS

### Effects of MAA fermentation supernatant on rice growth promotion

**Fig 2:**
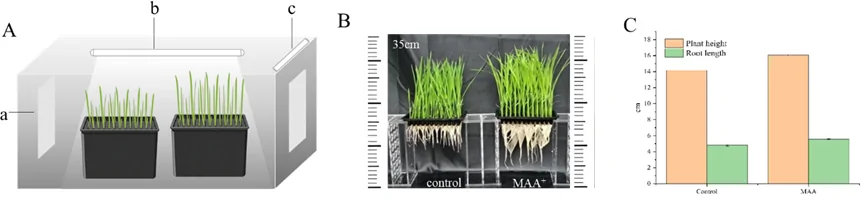
(**A**) Schematic diagram of hydroponic cultivation in a sterile environment. (a), gas exchange membrane; (b), plant supplementary lighting fixture; (c), ultraviolet lamp. (**B**) Growth-promoting effect of MAA on rice. (**C**) Effects of MAA treatment on shoot height and root length of rice seedlings.

The growth-promoting effects of MAA fermentation supernatant were evaluated using a hydroponic rice seedling system. As shown in Fig. 2, comparative analysis between the treatment group and the non-inoculated control group revealed significant morphological differences after 7 days of cultivation. Seedlings treated with MAA fermentation supernatant showed a 13.55% increase in shoot elongation compared to control (*P* < 0.05), reaching an average height of 16.08 cm versus 14.16 cm in the control. Simultaneously, primary roots exhibited 1.164 times greater elongation, and adventitious root formation was enhanced by 12.5% compared to control (*P* < 0.05).

### Effects of MAA on root endophytic fungal and bacterial communities

To investigate whether MAA could associate with rice roots and modulate the root-associated microbiome, we analyzed the endophytic fungal and bacterial communities in rice roots via ITS and 16S rRNA gene amplicon sequencing, respectively.

#### Effect of MAA on root endophytic fungal community

**Fig 3:**
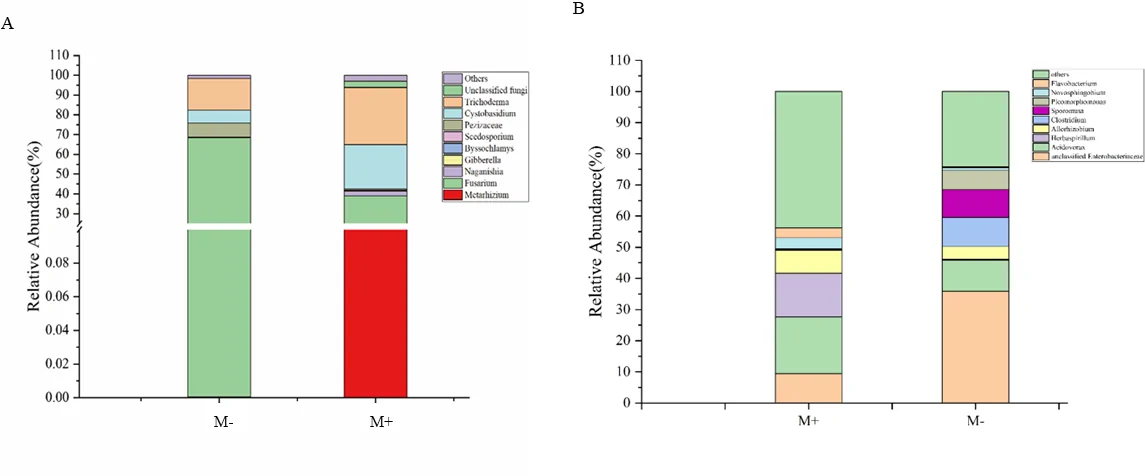
(**A**) Fungal community composition at the genus level. (**B**) Bacterial community composition at the genus level. Note: M+, rice roots collected from soil-based cultivation system inoculated with MAA; M−, untreated control.

The MAA treatment group (M^+^) exhibited a 400-fold increase in *Metarhizium* reads compared to the control group (M−) (from 0.00045% to 0.18%; *P* < 0.01) (Fig. 3A). As shown in Fig. 3A, the M^+^ group significantly altered the composition of the endophytic fungal community. Notably, the relative abundance of *Fusarium* sp.—a pathogenic genus associated with root rot—was markedly reduced by 76.15% (*P* < 0.05). Similarly, *Dictyostelium* sp. (chlorosis-associated) and *Pezizaceae* sp. (associated with chlorotic lesions) declined by 47.81% and 89.67%, respectively. Additionally, the MAA treatment inhibited the proliferation of *Gibberella* sp., a soilborne pathogen responsible for rice seedling blight. Conversely, the treated roots exhibited a significant increase in beneficial fungal genera: *Trichoderma* sp. (a plant growth-promoting taxon) and *Byssochlamys* sp. (known to suppress fungal pathogens) were enriched by 79.71% and 0.0032%, respectively (*P* < 0.01) (Fig. 3A).

#### Effect of MAA on root endophytic bacterial community

At the genus level, 16S rRNA gene sequencing revealed distinct bacterial community structures between MAA-treated (M^+^) and untreated (M⁻) samples (Fig. 3B). The relative abundance of unclassified Enterobacteriaceae decreased in the M^+^ group, while plant growth-promoting bacteria, including *Herbaspirillum* (27) and *Acidovorax* (28), were significantly enriched (Fig. 3B).

These findings indicate that MAA is strongly associated with the rice rhizosphere. MAA treatment restructured the composition of root-associated fungi and bacteria, enriching beneficial symbiotic and antagonistic microbial taxa while markedly suppressing pathogenic fungi within the root endophytic microbiota. However, as ITS sequencing cannot distinguish the inoculated *M. robertsii* ARSEF 23 strain from naturally occurring *Metarhizium* taxa, this evidence supports root association rather than unequivocal proof of colonization by the specific inoculated strain.

### Effects of culture conditions on MAA growth and rice growth promotion

**Fig 4:**
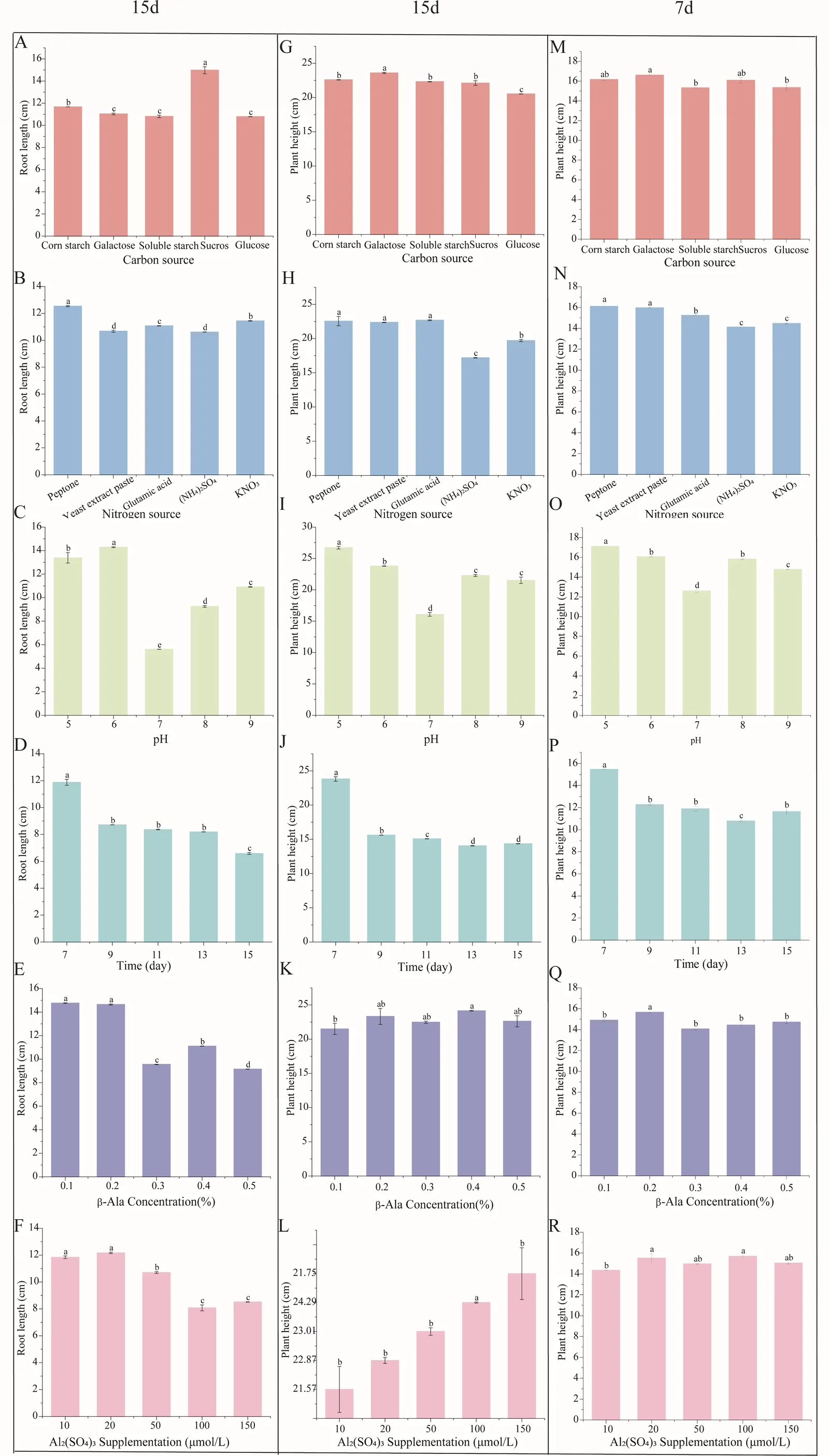
Growth responses of Rice seedlings to *M. robertsii* fermentation variables. (**A–F**) Root length under different treatments. (**A**) Carbon source; (**B**) Nitrogen source; (**C**) pH value; (**D**) Culture time (days); (**E**) β-Ala concentration (%); (**F**) Al₂(SO₄)₃ supplementation (μmol/L). (**G–L**) Total plant height under different treatments. (**G**) Carbon source; (**H**) Nitrogen source; (**I**) pH value; (**J**) Culture time (days); (**K**) β-Ala concentration (%); (**L**) Al₂(SO₄)₃ supplementation (μmol/L). (**M–R**) Shoot height under different treatments. (**M**) Carbon source; (**N**) Nitrogen source; (**O**) pH value; (**P**) Culture time (days); (**Q**) β-Ala concentration (%); (**R**) Al₂(SO₄)₃ supplementation (μmol/L). Different lowercase letters indicate significant differences among treatments (*P* < 0.05). Error bars: ±SE; different letters denote significant differences (*P* < 0.05).

To systematically evaluate the effects of culture conditions on MAA growth and its rice growth-promoting potential, we selected six critical variables [carbon source, nitrogen source, pH, cultivation time, β-Ala concentration, Al₂(SO₄)₃ supplementation] for optimization, which could critically influence fungal growth and metabolite production. Specifically, β-Ala may enhance the secretion of phytohormones and siderophores, influencing metabolic pathways in fungi (29). In plant-associated microbes, β-Ala derivatives have been implicated in stress tolerance and growth promotion. Al₂(SO₄)₃ may act synergistically to modulate metal ion availability, potentially affecting fungal metabolite secretion and nutrient acquisition. Using the basic fermentation medium as a control, the supplementation of sucrose and peptone as carbon and nitrogen sources increased plant height and root length by 24.59%, 22.55%, and 77.17%, 72.73%, respectively, which was superior to other treatment groups (*P* < 0.05) (Fig. 1). In addition, compared with the control group, supplementing 0.2% β-Ala resulted in an increase of 28.47% and 76.64% in plant height and root length, respectively (*P* < 0.01). The addition of 50 μmol/L Al₂(SO₄)₃ increased plant height and root length by 27.51% and 68.04%, respectively (Fig. 4A and B). After 7 days of cultivation in the fermentation broth, there were significant differences in plant height and root length between the hydroponic group and the control group, with increases of 29.97% and 71.19%, respectively. Furthermore, when the pH was adjusted to 6, plant height and root length increased by 29.86% and 75.54% compared to the base medium (Fig. 1A and B). These results collectively demonstrate significant variations in the plant-growth-promoting effects of MAA fermentation supernatant under different culture conditions.

The effects of different culture medium components on MAA growth were observed through colony morphology. The corresponding media compositions are listed in Table S4. Colony morphology exhibited significant variation across culture conditions (*P* < 0.05), with diameter measurements demonstrating clear media-dependent growth patterns (Fig. 1C). The numbers in parentheses indicate the numbering of culture media in supplementary materials list Table S4. The carbon source screening results showed that sucrose (10) supported maximum growth (diameter 15.82 cm), significantly better than other carbon sources. The colonies were circular with neat edges, and the surface of the colony was dry, presenting a dense fuzzy texture. Peptone (12) produced the largest colony diameter (15.50 cm) and a unique white mycelial morphology with abundant sporulation. The medium with 0.2% β-Ala produced the largest colonies (28) (17.73 cm), and the hyphae showed obvious spreading growth. The Al₂(SO₄)₃ tolerance test showed that when 100 μmol/L (20) was added, the colony size reached its maximum of 16.11 cm (Fig. 1C). For detailed information and descriptions, refer to Table S5. Systematic evaluation using standardized media formulations (Table S4) revealed significant culture-dependent variations in both MAA colony growth and rice growth promotion, and a positive correlation was observed between the extent of MAA growth stimulation and the efficacy in promoting rice growth. Based on these findings, key parameters including carbon source, nitrogen source, pH, fermentation duration, β-Ala concentration, and Al₂(SO₄)₃ supplementation were selected for single-factor optimization experiments to enhance the plant growth-promoting capacity of MAA.

### Single-factor test

The fermentation broth’s efficacy exhibited distinct response patterns to individual culture parameters. Fermentation time exhibited an inverse relationship with growth promotion. The 7 day treatment yielded the optimal plant height of 15.46 cm. In contrast, extended incubation to 15 days shifted biomass allocation toward shoot growth (23.82 cm) at the expense of root development (11.88 cm). β-Alanine concentration followed a biphasic trend, peaking at 0.2% with plant heights of 15.66 cm (7 days) and 14.65 cm (15 day roots)—representing 12.3% and 23.1% increases versus 0.1%, respectively (*P* < 0.05). Aluminum sulfate displayed concentration-specific regulation, where 100 μmol/L maximized plant height (15.69 cm at 7 days; 24.29 cm at 15 days), while 20 μmol/L optimized root development (11.9 cm). Nutrient sources differentially modulated growth: sucrose yielded maximum 15-day root elongation (14.98 cm), whereas peptone enhanced both plant height and root metrics (16.11 cm 7 day height; 12.54 cm 15 day roots). pH effects culminated at pH 6 with 23.79 cm root length, demonstrating narrow optimal ranges for metabolic activity.

### Response surface test optimization results

The response surface test’s design and results are shown in Table S2.

### Regression equation fitting and analysis of variance

A regression model for root length was established based on statistical analysis of the experimental data, as expressed in the following equation: Root length = 12.14 + 0.2739 × *a* + 0.0044 × *b* − 0.1472 × *c* − 0.0756 × *d* + 0.0875 × *ab* + 0.1888 × *ac* − 0.1000 × *ad* + 0.2038 × *bc* − 0.0200 × *bd* + 0.0688 × *cd* − 0.2243 × *a^2^* − 0.6093 × *b^2^* − 0.6643 × *c^2^* − 0.5793 × *d^2^*.

Variance analysis and a significant difference test were conducted for the regression model, and the results are shown in Table S3. The model was significant (F = 16.11, *P* < 0.0001), with model terms significant at *P* < 0.05. In this case, *a*, *ac*, *bc*, *b^2^*, *c^2^*, and *d^2^* are significant model terms. Values greater than 0.1000 indicate that the model terms are not significant. If there are many insignificant model terms (not counting those required to support hierarchy), model reduction may improve the model. The lack-of-fit F-value of 1.94 implies that the lack of fit is not significant relative to the pure error. There is a 24.12% chance that a lack-of-fit F-value this large could occur due to noise. A non-significant lack of fit is good—we want the model to fit. The equation in terms of coded factors can be used to make predictions about the response for given levels of each factor. By default, the high levels of the factors are coded as +1 and the low levels are coded as −1. The coded equation is useful for identifying the relative impact of the factors by comparing the factor coefficients.

### Response surface analysis of interaction of various factors

**Fig 5:**
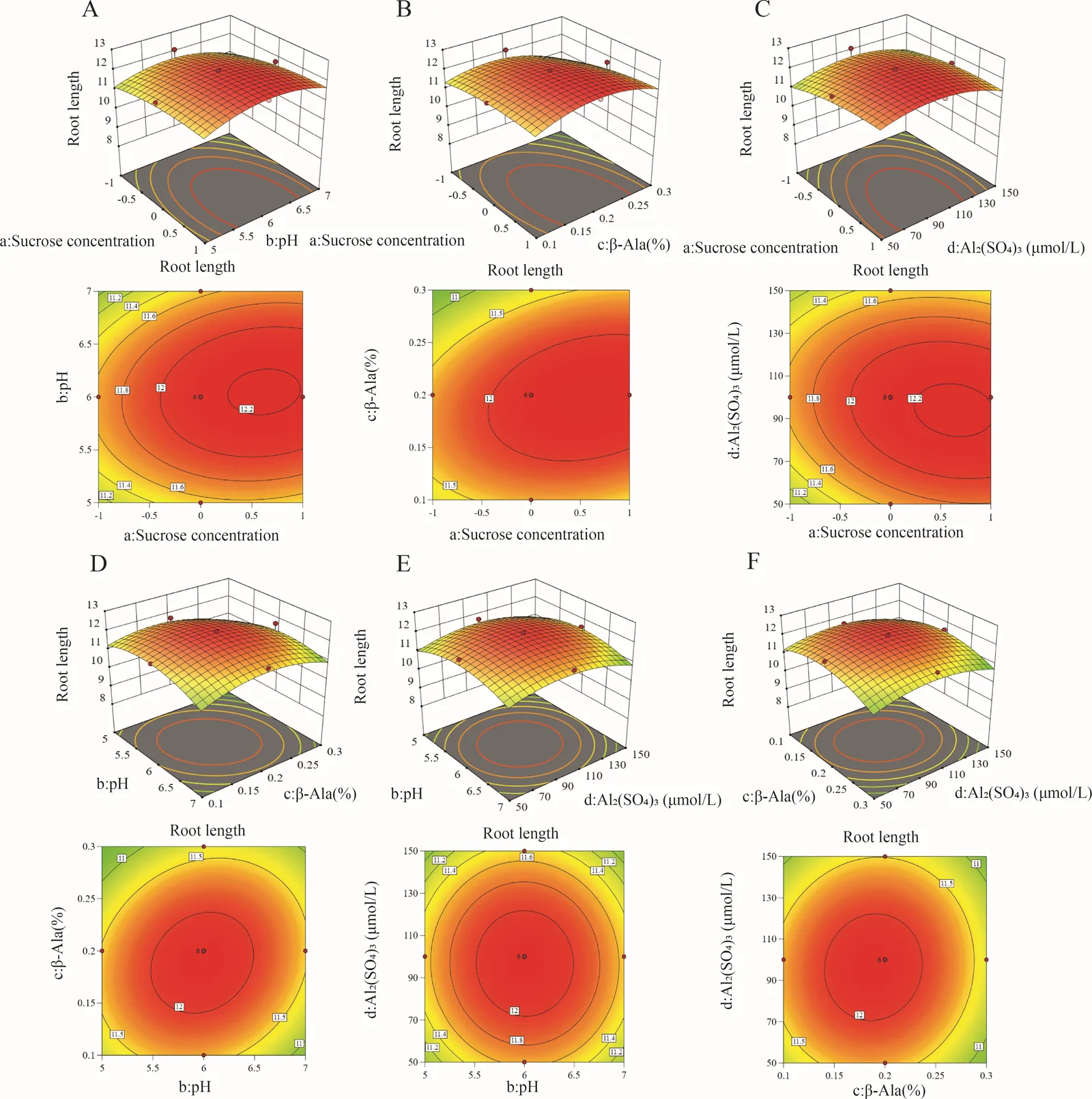
The response surface methodology and contour plots of the effects of the interaction between pH and sucrose concentration. Three-dimensional response surface plots and corresponding two-dimensional contour plots show pairwise interactions between variables. (**A**) Interaction between sucrose concentration (a) and pH (b); (**B**) Interaction between sucrose concentration (a) and β-Ala concentration (c); (**C**) Interaction between sucrose concentration (a) and Al₂(SO₄)₃ concentration (d); (**D**) Interaction between pH (b) and β-Ala concentration (c); (**E**) Interaction between pH (b) and Al₂(SO₄)₃ concentration (d); (**F**) Interaction between β-Ala concentration (c) and Al₂(SO₄)₃ concentration (d). Note: The incline degree of the surface diagram is directly proportional to the influence degree of factors on the response value. The larger the focal length of the contour, the stronger the interaction between parameters.

The response surface diagrams intuitively reflect the interactions between the key factors and their influence on rice root length. From the surface plots and contours in Fig. 5, the interactions between sucrose concentration (a), pH (b), β-Ala concentration (c), and Al₂(SO₄)₃ supplementation (d) and their significant effects on root length can be observed. This observation is consistent with the ANOVA results shown in Table S3, where interaction terms ac (sucrose concentration × β-Ala) and bc (pH × β-Ala) were identified as significant model terms (*P* < 0.05).

The optimal conditions were obtained using a quadratic polynomial regression fitting equation: sucrose concentration = 26 g/L; pH = 6.03; β-Ala concentration = 0.20%; and Al₂(SO₄)₃ supplementation = 96.07 µmol/L. Under these conditions, the predicted root length was 12.23 cm. For simple and feasible operation, the optimal fermentation conditions were determined as follows: sucrose concentration of 30 g/L, pH of 6.0, β-Ala concentration of 0.2%, and Al₂(SO₄)₃ concentration of 100 μmol/L.

### Establishment and validation of optimal culture conditions

The model regression equation was resolved using the software to derive the optimum culture conditions. The fermentation broth was prepared under the optimal fermentation conditions: sucrose concentration was 26 g/L, pH was 6.0, β-Ala 0.2%, and Al_2_(SO_4_)_3_ 100 μmol/L and repeated three times to verify the accuracy of the model. The average root length was 11.95 cm, which was consistent with the predicted value of 12.23 cm.

### Effect of different cultures on root hairs and SPAD in rice roots

In rice, root hairs constitute an essential part of the root system, contributing to nutrient and water uptake and transport, as well as plant anchorage (30). These functions underscore the critical role of root hairs in rice growth and development. SPAD values, which serve as an indicator of chlorophyll content, are widely used to assess photosynthetic efficiency in green plants (31). They reflect key physiological processes including plant growth and development, metabolic changes, and nutritional status, and are commonly employed as a reference parameter in environmental physiology studies. Consequently, chlorophyll content is frequently adopted as a physiological marker to investigate plant growth and development.

**Fig 6:**
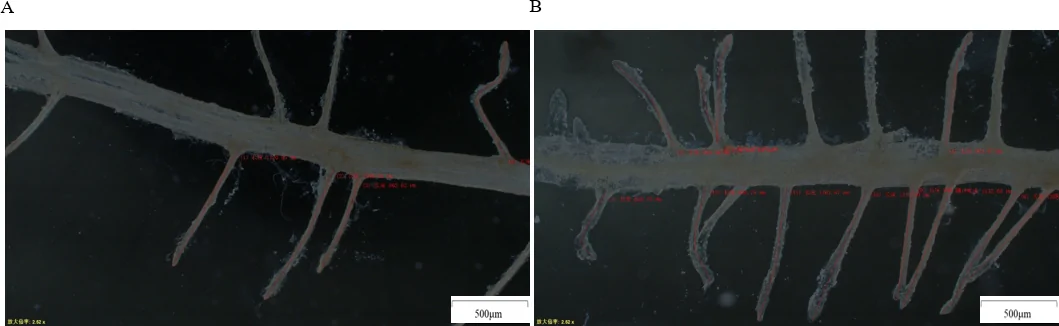
Effects of different MAA fermentation supernatants on rice root hair development. (**A**) Control: rice treated with supernatant from MAA basal medium after 7 days of fermentation. (**B**) Treatment: rice treated with supernatant from the optimal medium (sucrose, pH 6, 0.2% β-Ala, and 100 μmol/L Al₂(SO₄)₃) after 7 days.

**Fig 7:**
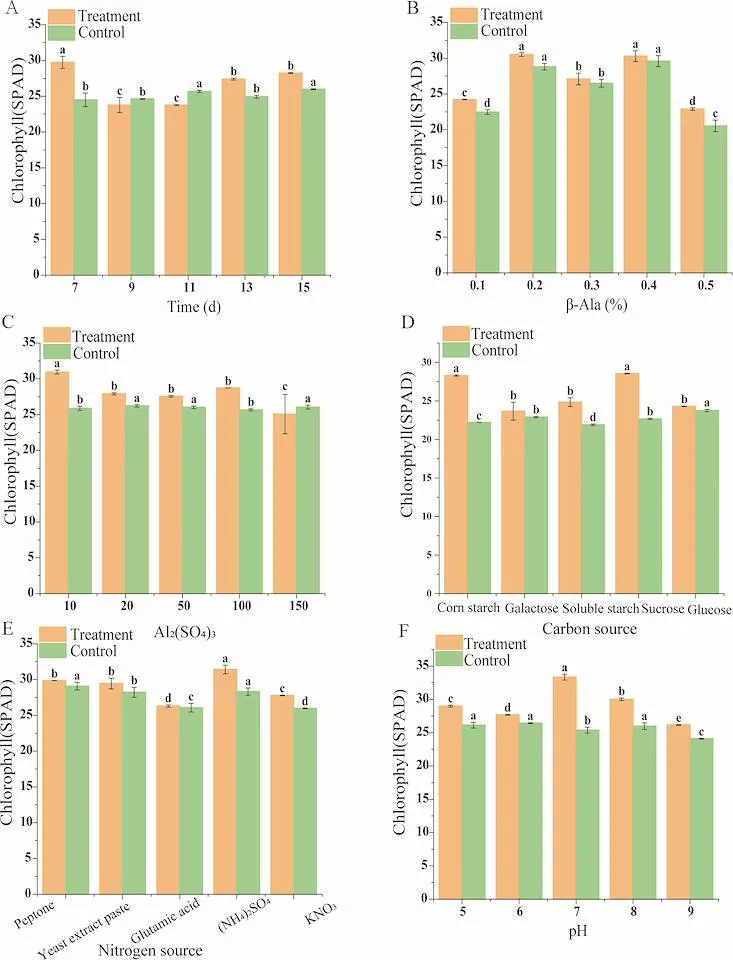
Effects of different cultures of fermentation time. (**A**) Culture time (days); (**B**) β‑Ala concentration (%); (**C**) Al₂(SO₄)₃ supplementation; (**D**) Carbon source; (**E**) Nitrogen source; (**F**) pH value. Different lowercase letters indicate significant differences among treatments (P<0.05).

Root hair morphology observations revealed distinct differences between the untreated control (Fig. 6A) and the optimal MAA treatment group (Fig. 6B) (selected based on an integrated assessment of all physiological parameters). As demonstrated in Fig. 6, plants treated with MAA exhibited significantly denser and longer root hairs compared to the blank control, indicating enhanced root system development and improved capacity for nutrient and water acquisition. SPAD values in rice plants treated with MAA fermentation broth initially decreased and then increased with prolonged fermentation time, showing the most pronounced effect and the highest chlorophyll reading (29.723) at 7 days of fermentation compared to the blank control (Fig. 7A). During the treatment period, SPAD values exhibited a trend of initial increase followed by a decrease with rising β-Ala concentration. The peak was observed at 0.2% β-Ala, with chlorophyll contents of 30.513 and 30.3 recorded at 0.2% and 0.4% β-Ala, respectively (*P* < 0.05) (Fig. 7B). As shown in Fig. 7C, the addition of Al₂(SO₄)₃ at various concentrations significantly enhanced chlorophyll content compared to the control, with the most notable effect at 10 μmol/L (chlorophyll content = 30.94), followed by 100 μmol/L (approximately 28.73). Furthermore, different carbon sources in the MAA culture differentially influenced chlorophyll levels, with corn flour and sucrose yielding the most marked effects (approximately 28.29 and 28.54, respectively). Variations in nitrogen sources also affected chlorophyll content, among which (NH₄)₂SO₄ resulted in the highest value (31.41). Finally, the pH of the fermentation broth significantly modulated chlorophyll accumulation, with pH 7 inducing a substantially greater response (chlorophyll content = 33.33) than other treatments.

In summary, these findings indicate that MAA fermentation duration, along with the concentrations of β-Ala and Al₂(SO₄)₃, the type of carbon and nitrogen sources, and the pH, all significantly regulate photosynthetic capacity and root system development in rice, as reflected by SPAD values and root hair morphology. Based on comprehensive evaluation, the optimal conditions for enhancing these key physiological and morphological traits were determined as follows: 7 days of fermentation, 0.2% β-Ala, 100 μmol/L Al₂(SO₄)₃, sucrose as the carbon source, peptone as the nitrogen source, and pH 7.

### Preliminary exploration of growth-promoting mechanisms based on phenotypic and microbial data

To gain insight into how the optimized MAA fermentation supernatant promotes rice growth, we integrated the phenotypic, physiological, and microbial community data obtained in this study to propose a dual-pathway mechanistic model.

Direct growth-promoting effects. Rice seedlings treated with the optimal MAA supernatant exhibited significantly denser and longer root hairs (Fig. 6) and a 21.7% increase in root length compared to the control (see the Section "Response Surface Test Optimization Results). These root architectural changes are consistent with auxin-mediated stimulation. Although we did not directly quantify IAA in the supernatant, the observed root hair phenotype, together with the fact that β-alanine (a precursor of coenzyme A) can enhance the biosynthesis of indole derivatives in fungi (32), suggests that MAA may secrete auxin-like compounds. Furthermore, the marked increase in chlorophyll content (SPAD value rose by 28.17% under optimal conditions, Fig. 7) indicates enhanced photosynthetic capacity. Higher chlorophyll levels may provide more photosynthates to support root and shoot growth, forming a positive feedback loop.

Indirect growth-promoting effects via rhizosphere fungal community restructuring. MAA was found to associate with rice roots, increasing its relative abundance by 400-fold (from 0.00045% to 0.18%, see the section Effects of MAA on Root Endophytic Fungal and Bacterial Communities). This strong root association was accompanied by a significant restructuring of the root endophytic fungal community. Notably, the relative abundance of the pathogenic genus *Fusarium* was reduced by 76.15%, while the plant growth-promoting genus *Trichoderma* was enriched by 79.71% (Fig. 3). Suppression of soilborne pathogens and enrichment of beneficial microbes may alleviate biotic stress and promote nutrient availability, thereby indirectly enhancing rice growth. This microbial community modulation may represent an important indirect pathway underlying the growth-promoting effect of MAA.

Role of optimized fermentation additives. The significant improvement in growth promotion under optimized conditions [pH 6.0, 0.2% β-Ala, 100 μmol/L Al₂(SO₄)₃] suggests that these additives may enhance the production of bioactive secondary metabolites. β-Alanine is a precursor of pantothenate and coenzyme A, and has been shown to trigger enzymes involved in the biosynthesis of phosphopantetheine, which is essential for the activation of multidomain enzymes responsible for secondary metabolite synthesis (29). Al₂(SO₄)₃ may act as a pH stabilizer and modulate metal ion availability, potentially influencing fungal metabolism. Together, these additives likely boost the secretion of growth-promoting compounds (e.g., auxin analogs, siderophores) by MAA, as reflected by the superior physiological responses of rice seedlings.

Collectively, these analyses based on existing data suggest that the growth-promoting effect of MAA on rice involves both direct (auxin-like stimulation and enhanced photosynthesis) and indirect (pathogen suppression and enrichment of beneficial microbes) mechanisms, with the optimized fermentation conditions further amplifying the production of bioactive metabolites. We acknowledge that direct molecular evidence (e.g., quantification of IAA, siderophores, or gene expression) is lacking, and these hypotheses need to be validated in future studies.

## DISCUSSION

This study is the first to confirm the direct growth-promoting effects of *Metarhizium robertsii* ARSEF 23 (MAA) and its fermented supernatant on rice, and we optimized the fermentation conditions of MAA for rice growth promotion by single-factor experiments combined with RSM. Hydroponic experiments confirmed that the optimized MAA fermentation supernatant could significantly improve rice root length, plant height, chlorophyll content, and root hair development, which not only verified the plant growth-promoting potential of MAA under hydroponic conditions but also provided a theoretical technical basis for the development of MAA-derived bio-inoculants for rice production pending soil and field validation.

The significant growth-promoting effect of MAA on rice is likely mediated, at least in part, by its association with the rice rhizosphere and possible root association, and root association is a hallmark of *Metarhizium*-plant interactions (33). ITS sequencing analysis revealed a 400-fold rise in *Metarhizium* reads within treated rice roots, suggesting strong root association, which may have contributed to subsequent growth promotion and pathogen suppression. The reshaping of the root fungal community by MAA—especially the enrichment of beneficial *Trichoderma* species and the suppression of pathogenic *Fusarium*—may represent an important indirect regulatory pathway for its growth-promoting effect. In addition, *Metarhizium* application significantly enhanced the proportion of beneficial bacteria in plant roots. This is consistent with the well-documented capacity of *Metarhizium* spp. to boost plant growth by optimizing the rhizosphere microbial environment (8). This microbial community reconstruction creates a more favorable growth environment for rice and may act in concert with the direct physiological promotion of MAA, contributing to the overall improvement of rice seedlings.

Prior research has shown that optimizing MAA’s culture conditions can greatly enhance its growth and effectiveness as a biocontrol fungus (1), and our study further verified optimizing the cultivation conditions to influence the metabolites of MAA and further enhance its promoting effect on rice growth. We identified that a sucrose concentration of 26 g/L and a pH of 6.0 were optimal for the growth of MAA. More importantly, our data indicated that adding specific compounds, including β-Ala and Al₂(SO₄)₃ supplementation, further boosted MAA’s biomass production and secondary metabolite synthesis. As previously suggested, β-Ala, as a precursor of coenzyme A, probably promoted the synthesis of growth-stimulating metabolites like indole derivatives (32), whereas Al₂(SO₄)₃ might function as a pH stabilizer and potentially influence metabolic activity, but these mechanisms remain hypothetical without direct metabolomic evidence. These results suggest that customized culture conditions can substantially improve MAA’s bioactivity, and the RSM-derived optimal conditions (pH 6.0, 0.20% β-Ala, 100 μmol/L Al₂(SO₄)₃) enhanced rice root length by 21.7% compared to the control, which provides a theoretical basis for a potentially scalable fermentation process for the future production of MAA biofertilizer pending validation at larger scales.

Hydroponic experiments demonstrated the biostimulatory potential of MAA fermentation broth, and the phenotypic enhancements of rice seedlings may stem from dual mechanisms. On the one hand, MAA improves the root-associated community by associating with the plant rhizosphere, increasing the proportion of beneficial microorganisms, and thereby indirectly promoting plant growth. On the other hand, although we did not quantitatively measure IAA or other phytohormones, previous studies have demonstrated that *Metarhizium* species can produce IAA and siderophores (20); fungal-derived auxin analogs directly stimulate cell elongation and root branching (34), which may be reflected in the significant increase of rice root length and the abundant development of root hairs in the treatment group. Meanwhile, MAA may promote nutrient solubilization via organic acid secretion, which facilitates phosphorus and iron uptake and further provides essential nutrients for chlorophyll synthesis and plant growth. Importantly, the inclusion of β-alanine in the optimized medium may hypothetically enhance the production of these bioactive metabolites, as β-alanine is a precursor of coenzyme A and can trigger the expression of enzymes involved in secondary metabolism (29); however, the actual metabolites responsible for the observed growth promotion remain unidentified, and this hypothesis awaits rigorous testing via untargeted metabolomics. A physiological correlation between root length and chlorophyll content was observed in rice seedlings: the 21.7% increase in root length may have enhanced the rice’s capacity for water and mineral nutrient uptake, which likely contributed to a 28.17% increase in chlorophyll content in the treatment group. Higher chlorophyll content improves photosynthetic efficiency and provides more photosynthates for root and shoot growth, forming a positive feedback loop that continuously promotes rice seedling growth. This physiological connection partly explains the rationality of selecting root length as the primary evaluation index, and root length, as a sensitive and early phenotypic indicator (30), can serve as a reliable indicator of the bioactivity of MAA fermentation supernatant on rice growth at the seedling stage.

Despite the significant findings, this study has certain limitations that need to be addressed in future research. First, the hydroponic system lacks soil-microbe complexity, potentially overestimating field efficacy (we will conduct pot and field experiments under diverse agroclimatic conditions to verify the effect on rice yield and evaluate its actual application potential). Second, while β-Ala improved fungal performance, its economic viability for large-scale fermentation requires techno-economic analysis, and we will explore cheaper alternative additives to reduce production costs. Third, although we have proposed dual mechanisms for the growth-promoting effect of MAA, direct molecular evidence—such as quantification of IAA, siderophores, or other secondary metabolites in the fermentation supernatant, as well as gene expression analysis of key biosynthetic pathways—is currently lacking. Therefore, the proposed mechanisms remain hypothetical and require further validation through targeted metabolomics and genetic approaches. Fourth, while we observed a 400-fold increase in the relative abundance of *Metarhizium* in treated roots using ITS amplicon sequencing, this method cannot distinguish between the inoculated *M. robertsii* ARSEF 23 strain and naturally occurring *Metarhizium* taxa. Therefore, our data provide strong evidence for root association, but not unequivocal proof of root association by the specific inoculated strain. Future studies should employ strain-specific markers (e.g., GFP tagging or selective primers) to unambiguously confirm root association and monitor its dynamics. Fifth, all experiments in this study were conducted under hydroponic conditions, which lack the physicochemical complexity and native microbial interactions of natural soil ecosystems. Therefore, the growth-promoting effects observed here may not directly translate to soil or field conditions. While hydroponic systems are valuable for mechanistic studies and fermentation optimization, future research must validate these findings under realistic agroecological conditions using pot and field trials across different soil types and climatic zones. Until such validation is performed, the applicability of our optimized MAA fermentation protocol should be considered as a theoretical basis rather than a ready-to-use field practice. Sixth, all fermentation optimization experiments in the present study were performed in shake-flask rather than instrumented bench-top or industrial fermenters. Although fermenters represent the standard platform for large-scale microbial production, the core objective of this work was to clarify whether key fermentation parameters [carbon/nitrogen sources, pH, temperature, β-Ala, and Al₂(SO₄)₃ supplementation] regulate the rice growth-promoting capacity of *M. robertsii*—a fundamental biological question that does not require fermenter-scale cultivation. Shake-flask systems are widely accepted for initial physiological screening and mechanistic hypothesis exploration in fungal-plant interaction research (11, 35). Nevertheless, the scale-up from shake flasks to controlled stirred-tank bioreactors is not straightforward; differences in oxygen transfer efficiency, fluid mixing pattern, and mechanical shear stress can substantially alter fungal physiological status and secondary metabolite biosynthesis. Accordingly, the optimal fermentation parameters obtained in this study should be regarded only as a foundational starting point for subsequent scale-up verification, rather than a directly transferable industrial production protocol. We are currently conducting further validation in a 5 L stirred-tank bioreactor to evaluate the robustness and applicability of these optimized conditions under precisely controlled fermentation environments. Seventh, in this study, exogenous addition of β-alanine and aluminum sulfate to the fermentation medium markedly enhanced the growth-promoting efficacy of *Metarhizium robertsii* toward rice. Nevertheless, the mechanistic basis behind this improvement remains speculative. Since we did not conduct metabolomic profiling such as LC-MS or GC-MS, it remains unclear whether the enhanced growth-promoting performance is attributed to β-alanine and aluminum sulfate per se, their metabolic derivatives, or other accompanying metabolic alterations in the fermentation broth and rice roots. β-alanine was supplemented owing to its function as a pantothenic acid precursor and its documented benefits for fungal stress tolerance and spore quality (32). Aluminum sulfate was mainly used as a pH stabilizer and a sulfate ion donor, which is indispensable for the synthesis of sulfur-containing amino acids and secondary metabolites in *Metarhizium* (20). Although our optimization assays verified that both additives improved fungal spore production and subsequent rice growth, we cannot differentiate their direct effects (e.g., β-alanine as a signaling molecule) from indirect ones (e.g., redirecting fungal metabolic flux to synthesize growth-promoting compounds such as IAA and siderophores). We recognize this as a key limitation of the present work. Further research incorporating targeted or untargeted metabolomics of fermentation filtrates and rice root exudates, together with bioassay-guided fractionation, is required to characterize the functional metabolites. Until solid metabolomic evidence is obtained, the regulatory roles of β-alanine and aluminum sulfate proposed herein should be regarded only as preliminary hypotheses rather than definitive mechanisms. Eighth, due to limited biological replication for the pot experiments, multivariate analyses such as PCoA and PERMANOVA could not be reliably performed. This should be considered when interpreting the community-level comparisons. Additionally, co-inoculation trials with arbuscular mycorrhizal fungi should be carried out in future research to balance pathogen suppression and nutrient uptake synergies.

In conclusion, our study systematically optimized the fermentation conditions of MAA for rice growth promotion by RSM, and the optimized fermentation supernatant significantly improved rice root length, chlorophyll content, and root hair development. The growth-promoting effect of MAA may involve dual mechanisms of direct physiological regulation (auxin secretion and nutrient solubilization) and indirect microbial community reconstruction (pathogen suppression and beneficial microbe enrichment), which provides a preliminary framework for understanding the interaction between MAA and rice seedlings. These findings provide a theoretical basis for the development of MAA-derived bio-inoculants under optimized fermentation conditions, but their effectiveness in soil and field settings remains to be validated in future studies. The fermentation optimization strategy in this study also provides a reference for the fermentation improvement of other plant growth-promoting fungi. These findings not only contribute to the theoretical understanding of MAA–rice interactions but also offer a technical framework for sustainable rice production.

### Conclusion

This study has successfully identified *Metarhizium robertsii* as a viable plant growth-promoting agent for rice. Through optimizing key fermentation conditions [carbon/nitrogen sources, pH, β-Ala, and Al₂(SO₄)₃] via the Response Surface Methodology, we formulated a fermentation supernatant that significantly improves rice root length (by 21.7%), chlorophyll levels, and root system growth. Moreover, we propose that the growth-promoting effects may involve both direct physiological stimulation and indirect modulation of the root mycobiome, based on phenotypic and community-level evidence—specifically inhibiting pathogenic microbes while fostering beneficial fungal taxa. Our results offer an optimized fermentation protocol and mechanistic understanding, highlighting *M. robertsii* as a viable candidate for developing innovative microbial inoculants in sustainable rice farming.

## Data Availability

Data will be made available on request.

## SUPPLEMENTAL MATERIAL

Tables S1 to S5; Figure S1 spectrum.01009-26-s0001.docx Table S1: Codes and levels of factors for response surface methodology experiments. Table S2: Experimental design and results of strain culture conditions. Table S3: Regression analysis of experimental results based on Box-Behnken design. Table S4: Formulations of experimental media with varying carbon sources, nitrogen sources, pH, and additives. Table S5: The influence of different medium components on the colony growth of Fig. S1. Fig. S1: Single-factor experiment rice growth difference diagram.

ASM does not own the copyrights to Supplemental Material that may be linked to, or accessed through, an article. The authors have granted ASM a non-exclusive, world-wide license to publish the Supplemental Material files. Please contact the corresponding author directly for reuse.
