# Supplementary material for: Optimization of fermentation conditions for Metarhizium robertsii and its effect on rice growth
Source: Microbiol Spectr. 2026 Sep 24;14(11):e01009-26. doi: 10.1128/spectrum.01009-26 (PMC13729165; doi:10.1128/spectrum.01009-26)
Supplement: Supplementary file 1 [file spectrum.01009-26-s0001.docx]

**Table S1**

Table S1. Codes and levels of factors for response surface methodology experiments

| Variable | Factor | Factor level | | | |
| --- | --- | --- | --- | --- | --- |
|  |  | -1 | | 0 | 1 |
| A  B  C  D | Sucrose Concentration pH  β-Ala（%）  Al_2_（SO_4_）_3_（μmol/L） | | 10  5  0.1  50 | 20  6  0.2  100 | 30  7  0.3  150 |

**Table S2**

Table S2. Experimental design and results of strain culture conditions

| **Run** | **Factor** | | | |  |
| --- | --- | --- | --- | --- | --- |
|  | **A** | **B** | **C** | **D** | **Root length** |
|  | **Carbon source** | **pH** | **β-Ala** | **Al_2_SO_4_** |  |
| 1 | -1 | 5 | 0.1 | 150 | 10.17 |
| 2 | -1 | 5 | 0.1 | 50 | 10.65 |
| 3 | 1 | 5 | 0.1 | 150 | 10.52 |
| 4 | 0 | 6 | 0.2 | 100 | 11.99 |
| 5 | 0 | 6 | 0.3 | 100 | 11.69 |
| 6 | 1 | 7 | 0.3 | 50 | 10.57 |
| 7 | 0 | 6 | 0.2 | 100 | 12.08 |
| 8 | 0 | 7 | 0.2 | 100 | 11.72 |
| 9 | 0 | 6 | 0.2 | 150 | 11.57 |
| 10 | -1 | 5 | 0.3 | 50 | 8.9 |
| 11 | -1 | 7 | 0.1 | 150 | 9.84 |
| 12 | 0 | 6 | 0.2 | 100 | 11.44 |
| 13 | 1 | 5 | 0.3 | 50 | 10.47 |
| 14 | 0 | 6 | 0.2 | 100 | 12.23 |
| 15 | -1 | 6 | 0.2 | 100 | 12.08 |
| 16 | -1 | 7 | 0.3 | 150 | 9.53 |
| 17 | 1 | 5 | 0.3 | 150 | 9.73 |
| 18 | 1 | 7 | 0.3 | 150 | 10.7 |
| 19 | -1 | 7 | 0.3 | 50 | 9.53 |
| 20 | 1 | 5 | 0.1 | 50 | 10.3 |
| 21 | -1 | 5 | 0.3 | 150 | 9.56 |
| 22 | 0 | 5 | 0.2 | 100 | 11.72 |
| 23 | -1 | 7 | 0.1 | 50 | 9.72 |
| 24 | 1 | 6 | 0.2 | 100 | 12.13 |
| 25 | 0 | 6 | 0.2 | 100 | 11.89 |
| 26 | 0 | 6 | 0.2 | 100 | 12.11 |
| 27 | 1 | 7 | 0.1 | 50 | 10.7 |
| 28 | 1 | 7 | 0.1 | 150 | 9.79 |
| 29 | 0 | 6 | 0.2 | 50 | 11.93 |
| 30 | 0 | 6 | 0.1 | 100 | 11.64 |

**Table S3**

| **Source** | **Sum of Squares** | **DF** | **Mean Square** | **F-value** | **p-value** |  |
| --- | --- | --- | --- | --- | --- | --- |
| Model | 28.28 | 14 | 2.02 | 16.11 | < 0.0001 | significant |
| A-Carbon source | 1.35 | 1 | 1.35 | 10.77 | 0.0050 |  |
| B-pH | 0.0004 | 1 | 0.0004 | 0.0028 | 0.9582 |  |
| C-β-Ala | 0.3901 | 1 | 0.3901 | 3.11 | 0.0981 |  |
| D-Al_2_(S0_4_)_3_ | 0.1028 | 1 | 0.1028 | 0.8196 | 0.3796 |  |
| AB | 0.1225 | 1 | 0.1225 | 0.9770 | 0.3386 |  |
| AC | 0.5700 | 1 | 0.5700 | 4.55 | 0.0499 |  |
| AD | 0.1600 | 1 | 0.1600 | 1.28 | 0.2764 |  |
| BC | 0.6642 | 1 | 0.6642 | 5.30 | 0.0361 |  |
| BD | 0.0064 | 1 | 0.0064 | 0.0510 | 0.8243 |  |
| CD | 0.0756 | 1 | 0.0756 | 0.6032 | 0.4494 |  |
| A^2^ | 0.1303 | 1 | 0.1303 | 1.04 | 0.3241 |  |
| B^2^ | 0.9619 | 1 | 0.9619 | 7.67 | 0.0143 |  |
| C^2^ | 1.14 | 1 | 1.14 | 9.12 | 0.0086 |  |
| D^2^ | 0.8695 | 1 | 0.8695 | 6.93 | 0.0188 |  |
| Residual | 1.88 | 15 | 0.1254 |  |  |  |
| Lack of Fit | 1.49 | 10 | 0.1495 | 1.94 | 0.2412 | not significant |
| Pure Error | 0.3859 | 5 | 0.0772 |  |  |  |
| Cor Total | 30.16 | 29 |  |  |  |  |

Table S3. Regression analysis of experimental results based on Box-Behnken design

**Table S4**.

Table S4. Formulations of experimental media with varying carbon sources, nitrogen sources, pH, and additives

|  | **ID** | **Carbon source** | | | | | **Nitrogen source** | | | | | **Time(d)** | | | | | | **pH** | | | | | | **β-Ala(%)** | | | | | | **Al_2_(SO_4_)_3_(μmol/L)** | | | | |
| --- | --- | --- | --- | --- | --- | --- | --- | --- | --- | --- | --- | --- | --- | --- | --- | --- | --- | --- | --- | --- | --- | --- | --- | --- | --- | --- | --- | --- | --- | --- | --- | --- | --- | --- |
|  |  | **glucose** | **soluble starch** | **corn flour** | **galactose** | **sucrose** | **peptone** | **yeast** | **glutamic acid** | **(NH_4)2_SO_4_** | **KNO_3_** | **7** | **9** | **11** | **13** | **15** | **5** | | **6** | **7** | **8** | **9** | **0.1** | | **0.2** | **0.3** | **0.4** | **0.5** | **10** | | **20** | **50** | **100** | **150** |
| Control | 1 |  | √ |  |  |  | √ |  |  |  |  | √ |  |  |  |  |  | | √ |  |  |  |  | | √ |  |  |  |  | | √ |  |  |  |
|  | 2 |  |  |  |  | √ |  |  | √ |  |  | √ |  |  |  |  |  | | √ |  |  |  |  | | √ |  |  |  |  | | √ |  |  |  |
|  | 3 |  |  |  |  | √ | √ |  |  |  |  | √ |  |  |  |  |  | | √ |  |  |  |  | | √ |  |  |  |  | | √ |  |  |  |
|  | 4 |  |  |  |  | √ | √ |  |  |  |  | √ |  |  |  |  |  | | √ |  |  |  |  | | √ |  |  |  |  | |  | √ |  |  |
|  | 5 |  |  |  |  | √ | √ |  |  |  |  | √ |  |  |  |  |  | | √ |  |  |  | √ | |  |  |  |  |  | | √ |  |  |  |
|  | 6 |  |  |  |  | √ | √ |  |  |  |  | √ |  |  |  |  |  | | √ |  |  |  |  | | √ |  |  |  |  | | √ |  |  |  |
| Carbon source | 7 | √ |  |  |  |  | √ |  |  |  |  | √ |  |  |  |  |  | | √ |  |  |  |  | | √ |  |  |  |  | | √ |  |  |  |
|  | 8 |  | √ |  |  |  | √ |  |  |  |  | √ |  |  |  |  |  | | √ |  |  |  |  | | √ |  |  |  |  | | √ |  |  |  |
|  | 9 |  |  | √ |  |  | √ |  |  |  |  | √ |  |  |  |  |  | | √ |  |  |  |  | | √ |  |  |  |  | | √ |  |  |  |
|  | 10 |  |  |  | √ |  | √ |  |  |  |  | √ |  |  |  |  |  | | √ |  |  |  |  | | √ |  |  |  |  | | √ |  |  |  |
|  | 11 |  |  |  |  | √ | √ |  |  |  |  | √ |  |  |  |  |  | | √ |  |  |  |  | | √ |  |  |  |  | | √ |  |  |  |
| Nitrogen source | 12 |  |  |  |  | √ | √ |  |  |  |  | √ |  |  |  |  |  | | √ |  |  |  |  | | √ |  |  |  |  | | √ |  |  |  |
|  | 13 |  |  |  |  | √ |  | √ |  |  |  | √ |  |  |  |  |  | | √ |  |  |  |  | | √ |  |  |  |  | | √ |  |  |  |
|  | 14 |  |  |  |  | √ |  |  | √ |  |  | √ |  |  |  |  |  | | √ |  |  |  |  | | √ |  |  |  |  | | √ |  |  |  |
|  | 15 |  |  |  |  | √ |  |  |  | √ |  | √ |  |  |  |  |  | | √ |  |  |  |  | | √ |  |  |  |  | | √ |  |  |  |
|  | 16 |  |  |  |  | √ |  |  |  |  | √ | √ |  |  |  |  |  | | √ |  |  |  |  | | √ |  |  |  |  | | √ |  |  |  |
| Time | 17 |  |  |  |  | √ | √ |  |  |  |  | √ |  |  |  |  |  | | √ |  |  |  |  | | √ |  |  |  |  | | √ |  |  |  |
|  | 18 |  |  |  |  | √ | √ |  |  |  |  |  | √ |  |  |  |  | | √ |  |  |  |  | | √ |  |  |  |  | | √ |  |  |  |
|  | 19 |  |  |  |  | √ | √ |  |  |  |  |  |  | √ |  |  |  | | √ |  |  |  |  | | √ |  |  |  |  | | √ |  |  |  |
|  | 20 |  |  |  |  | √ | √ |  |  |  |  |  |  |  | √ |  |  | | √ |  |  |  |  | | √ |  |  |  |  | | √ |  |  |  |
|  | 21 |  |  |  |  | √ | √ |  |  |  |  |  |  |  |  | √ |  | | √ |  |  |  |  | | √ |  |  |  |  | | √ |  |  |  |
| pH | 22 |  |  |  |  | √ | √ |  |  |  |  |  |  |  |  |  | √ | | √ |  |  |  |  | | √ |  |  |  |  | | √ |  |  |  |
|  | 23 |  |  |  |  | √ | √ |  |  |  |  |  |  |  |  |  |  | | √ |  |  |  |  | | √ |  |  |  |  | | √ |  |  |  |
|  | 24 |  |  |  |  | √ | √ |  |  |  |  |  |  |  |  |  |  | |  | √ |  |  |  | | √ |  |  |  |  | | √ |  |  |  |
|  | 25 |  |  |  |  | √ | √ |  |  |  |  |  |  |  |  |  |  | |  |  | √ |  |  | | √ |  |  |  |  | | √ |  |  |  |
|  | 26 |  |  |  |  | √ | √ |  |  |  |  |  |  |  |  |  |  | |  |  |  | √ |  | | √ |  |  |  |  | | √ |  |  |  |
| β-Ala | 27 |  |  |  |  | √ | √ |  |  |  |  |  |  |  |  |  |  | | √ |  |  |  | √ | | √ |  |  |  |  | | √ |  |  |  |
|  | 28 |  |  |  |  | √ | √ |  |  |  |  |  |  |  |  |  |  | | √ |  |  |  |  | | √ |  |  |  |  | | √ |  |  |  |
|  | 29 |  |  |  |  | √ | √ |  |  |  |  |  |  |  |  |  |  | | √ |  |  |  |  | |  | √ |  |  |  | | √ |  |  |  |
|  | 30 |  |  |  |  | √ | √ |  |  |  |  |  |  |  |  |  |  | | √ |  |  |  |  | |  |  | √ |  |  | | √ |  |  |  |
|  | 31 |  |  |  |  | √ | √ |  |  |  |  |  |  |  |  |  |  | | √ |  |  |  |  | |  |  |  | √ |  | | √ |  |  |  |
| Al_2_(SO_4_)_3_ | 32 |  |  |  |  | √ | √ |  |  |  |  |  |  |  |  |  |  | | √ |  |  |  |  | |  |  |  |  | √ | | √ |  |  |  |
|  | 33 |  |  |  |  | √ | √ |  |  |  |  |  |  |  |  |  |  | | √ |  |  |  |  | |  |  |  |  |  | | √ |  |  |  |
|  | 34 |  |  |  |  | √ | √ |  |  |  |  |  |  |  |  |  |  | | √ |  |  |  |  | |  |  |  |  |  | |  | √ |  |  |
|  | 35 |  |  |  |  | √ | √ |  |  |  |  |  |  |  |  |  |  | | √ |  |  |  |  | |  |  |  |  |  | |  |  | √ |  |
|  | 36 |  |  |  |  | √ | √ |  |  |  |  |  |  |  |  |  |  | | √ |  |  |  |  | |  |  |  |  |  | |  |  |  | √ |

**Table S5**

Table S5. The influence of different medium components on the colony growth of

|  | ID | Colony diameter（mm） | Sporulation time | Neat edges | Dense hypha | Colony Shape |
| --- | --- | --- | --- | --- | --- | --- |
| Carbon source | 7 | 12.2122 | ＋＋ | Yes | Yes | Circular |
|  | 8 | 12.015 | ＋＋ | Yes | Yes | Spreading |
|  | 9 | 12.9004 | ＋＋ | Yes | Yes | Circular |
|  | 10 | 15.8202 | ＋＋＋＋＋ | Yes | Yes | Circular |
|  | 11 | 12.8 | ＋＋ | Yes | Yes | Circular |
| Nitrogen source | 12 | 15.5081 | ＋＋＋＋ | Yes | Yes | Circular |
|  | 13 | 15.4003 | ＋＋＋＋ | Yes | Yes | Punctiform |
|  | 14 | 10.4043 | ＋ | No | Yes | Irregular |
|  | 15 | 10.6005 | ＋ | No | No | Irregular |
|  | 16 | 14.6277 | ＋＋＋＋ | Yes | No | Spreading |
| β-Ala | 27 | 13.3454 | ＋＋＋ | Yes | Yes | Circular |
|  | 28 | 17.737 | ＋＋＋＋＋ | Yes | Yes | Spreading |
|  | 29 | 15.7153 | ＋＋＋＋＋ | Yes | Yes | Circular |
|  | 30 | 16.4438 | ＋＋＋＋＋ | Yes | Yes | Circular |
|  | 31 | 15.4208 | ＋＋＋＋＋ | Yes | Yes | Circular |
| Al_2_(SO_4_)_3_ | 32 | 14.0057 | ＋＋＋＋ | Yes | Yes | Spreading |
|  | 33 | 12.4064 | ＋＋ | Yes | Yes | Circular |
|  | 34 | 13.6092 | ＋＋＋ | Yes | Yes | Circular |
|  | 35 | 16.1112 | ＋＋＋＋＋ | Yes | Yes | Circular |
|  | 36 | 14.0228 | ＋＋＋ | Yes | Yes | Circular |

**Figure S1**

Figure S1. Single-factor experiment rice growth difference diagram.Figure caption: 1-5 represent five different factor levels. Under each factor, the left side of the rice is the control group and the right side is the experimental group.
